# Do Termites Avoid Carcasses? Behavioral Responses Depend on the Nature of the Carcasses

**DOI:** 10.1371/journal.pone.0036375

**Published:** 2012-04-27

**Authors:** Kok-Boon Neoh, Beng-Keok Yeap, Kunio Tsunoda, Tsuyoshi Yoshimura, Chow-Yang Lee

**Affiliations:** 1 Urban Entomology Laboratory, Vector Control Research Unit, School of Biological Sciences, Universiti Sains Malaysia, Penang, Malaysia; 2 Research Institute for Sustainable Humanosphere, Kyoto University, Uji, Kyoto, Japan; University of Osnabrueck, Germany

## Abstract

**Background:**

Undertaking behavior is a significant adaptation to social life in enclosed nests. Workers are known to remove dead colony members from the nest. Such behavior prevents the spread of pathogens that may be detrimental to a colony. To date, little is known about the ethological aspects of how termites deal with carcasses.

**Methodology and Principal Findings:**

In this study, we tested the responses to carcasses of four species from different subterranean termite taxa: *Coptotermes formosanus* Shiraki and *Reticulitermes speratus* (Kolbe) (lower termites) and *Microcerotermes crassus* Snyder and *Globitermes sulphureus* Haviland (higher termites). We also used different types of carcasses (freshly killed, 1-, 3-, and 7-day-old, and oven-killed carcasses) and mutilated nestmates to investigate whether the termites exhibited any behavioral responses that were specific to carcasses in certain conditions. Some behavioral responses were performed specifically on certain types of carcasses or mutilated termites. *C. formosanus* and *R. speratus* exhibited the following behaviors: (1) the frequency and time spent in antennating, grooming, and carcass removal of freshly killed, 1-day-old, and oven-killed carcasses were high, but these behaviors decreased as the carcasses aged; (2) the termites repeatedly crawled under the aging carcass piles; and (3) only newly dead termites were consumed as a food source. In contrast, *M. crassus* and *G. sulphureus* workers performed relatively few behavioral acts. Our results cast a new light on the previous notion that termites are necrophobic in nature.

**Conclusion:**

We conclude that the behavioral response towards carcasses depends largely on the nature of the carcasses and termite species, and the response is more complex than was previously thought. Such behavioral responses likely are associated with the threat posed to the colony by the carcasses and the feeding habits and nesting ecology of a given species.

## Introduction

Undertaking behavior is an essential adaptation in the evolution of eusocial insects. Insects use many sophisticated behaviors when they encounter carcasses in their nest to prevent the spread of pathogens that may be unfavorable to a colony. Necrophoresis is one of the common responses exhibited by ants and bees that inhabit large and enclosed nests. Such behavior was first described in the ant *Pogonomyrmex badius* (Latreille) [1]. The ant carcasses were picked up and carried away from the nest toward the refuse piles after being investigated by healthy workers [1]. This behavior has been reported in many ant species, including *Solenopsis invicta* Buren [1], *Myrmecia vindex* Smith [2], and *Atta mexicana* (F. Smith) [3]. In a honey bee (*Apis mellifera* Linn.) colony, 1–2% of the colony population work as undertakers, grasping and pulling dead nestmates to the nest exit and flying away to drop them 10 to 100 m from the nest [4].

In 1982, Su et al. [5] reported that the subterranean termite *Coptotermes formosanus* Shiraki avoided contact with dead termites that were killed by a slow-acting insecticide by sealing off the tunnel that provided access to the treated zone. Since that report, researchers have believed that termites are necrophobic in nature. However, this premise requires further investigation because conflicting data exist. Although many previous studies [6], [7], [8], [9] reported similar observations, the existence of such behavior often was inadequately explained [10], [11], [12]. Moreover, carcass-burying behavior [13], [14] and cannibalism [15] are common in termites when they encounter dead nestmates. In a recent study, various fatty acids, indole, and phenol were identified as eliciting the act of entombment in *Pseudacanthotermes spiniger* (Sjödtedt) dealates [16]. Other studies reported that when in contact with diseased or injured termites, alarming and allogrooming responses were performed by healthy nestmates [17], [18]. Unlike many social insects, necrophoresis is rarely demonstrated in termites. However, to date, little is known about the ethology of termites when they encounter dead nestmates.

In ants, the chemical signal from the carcasses (necromone) is known to elicit undertaking behavior [1], [2], [3], [19]. However, the resulting behavior depends on the nature of the carcasses. For example, in a study of the ant *Temnothorax lichtensteini* (Bondroit), the workers were found to bury newly dead carcasses but remove aged carcasses from their nests [20]. To date it is not known whether termites also behave differently when exposed to different ages or types of termite carcasses.

In this study, four termite species from different subterranean termite taxa were studied: *C. formosanus*, *Reticulitermes speratus* (Kolbe), *Microcerotermes crassus* Snyder, and *Globitermes sulphureus* Haviland. These four species were selected based on their biological differences. For example, *C. formosanus* and *R. speratus* are lower termites. They feed on wood and nest underground. *M. crassus* and *G. sulphureus* are higher termites that build mounds and forage for leaf and wood litter. Different aged carcasses and mutilated live workers, which might replicate the natural context (e.g., aggression interaction between termites; a diseased colony; predator invasion; accidental nest and foraging trail damage), were used to test the reaction of the termites to exposure to carcasses. We hypothesized that termites would perform a complex range of undertaking behaviors based on the nature of the carcasses and the feeding habits and nesting ecology of the particular termite species being tested.

## Results

Irrespective of the termite species, the worker termites responded in one universal manner towards nestmate carcasses when the carcasses were first introduced into the arena: As soon as the presence of the carcasses or mutilated nestmates was detected by workers, the workers immediately began to evacuate the area, heading to the access tubes, and then they antennated other non-exposed workers to signal and recruit them to the container in which the carcasses were located. The intervals between when termites discovered carcasses and evacuated and when termites were recruited to re-examine carcasses varied among the four species tested. On average, *C. formosanus* returned to the arena most quickly (±5 min), followed by *R. speratus* (±15 min), *M. crassus* (≥30 min), and *G. sulphureus* (≥30 min). This phenomenon was absent when healthy termites were introduced into the control sets. On many occasions, the introduced healthy termites were antennated and actively groomed by the resident termites, and alarming jerking behavior was occasionally exhibited by *M. crassus* and *G. sulphureus*.

**Figure 1:**
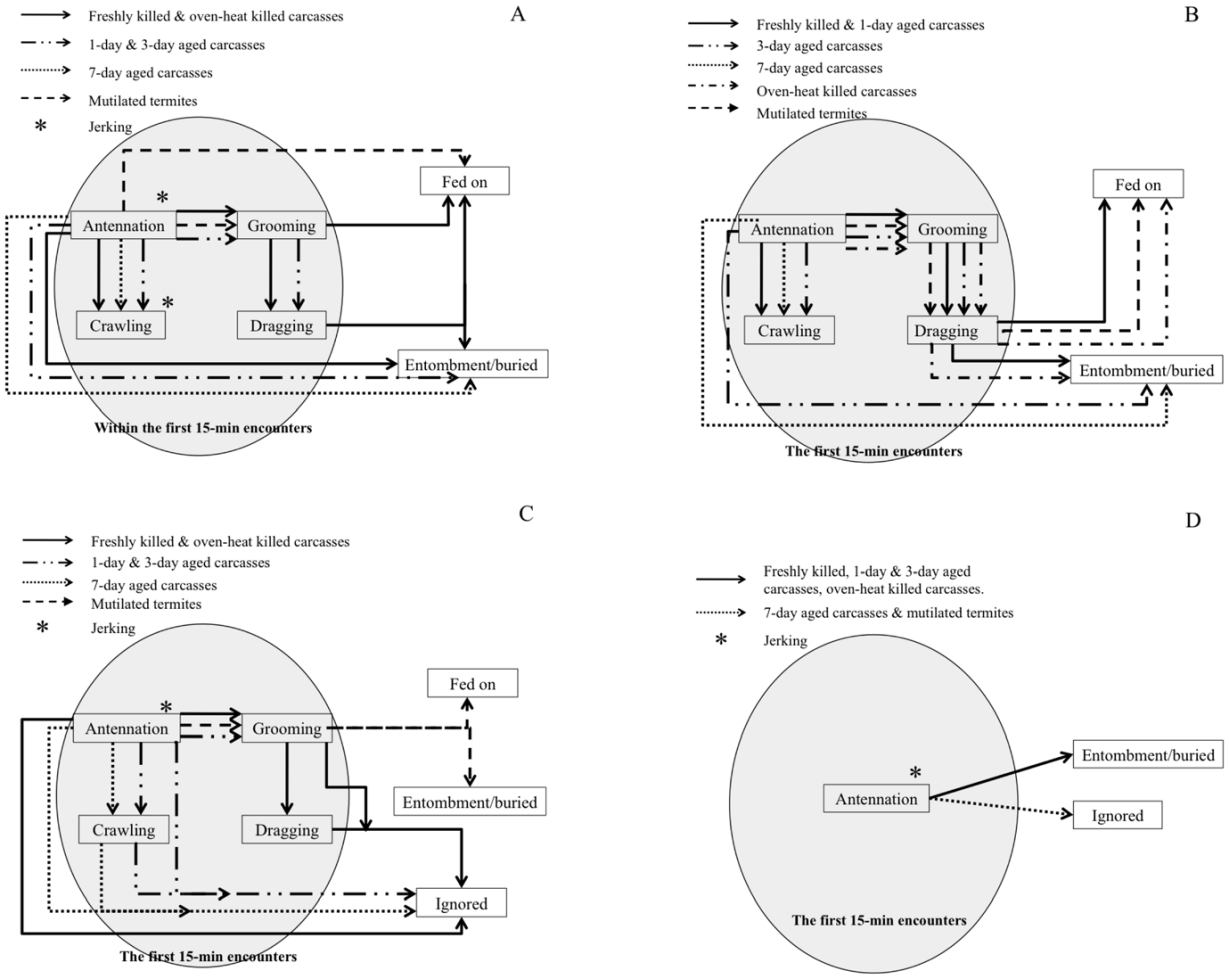
Behavioral repertoires of (A) *Coptotermes formosanus*, (B) *Reticulitermes speratus*, (C) *Microcerotermes crassus*, and (D) *Globitermes sulphureus* toward aged dead bodies, oven-killed bodies, and mutilated termites. * jerking may happen before/after antennating or crawling under dead piles. Ethograms illustrate the behaviors shown during the 15 min observation period beginning with the first contact (circled) and the way termites handled the carcasses after 48 h.

During the 15 min observation period, *C. formosanus* and *R. speratus* typically shared common repertoires of behavior when encountering carcasses or mutilated termites: antennation→crawling under carcass piles and antennation→grooming and antennation→grooming→dragging (Figure 1). However, *M. crassus* and *G. sulphureus* usually ignored or fled from the carcasses (Figure 1). We also observed distinct responses of worker termites to aged carcasses and mutilated termites. The behavior of worker termites encountering carcasses and mutilated termites and the frequency and the time spent in each behavior varied among the termite species tested (see below for each species).

### *C. formosanus*

There were significant differences in frequency and time spent in antennation among the different types of termite carcasses encountered (frequency: *F* = 26.233; df = 6, 14; *P*<0.01; time: *F* = 10.380; df = 6, 14; *P*<0.01). As shown in Table 1, The HSD post-hoc test revealed that workers were more inclined to explore (antennation) the freshly killed, 1-day-old, and oven-killed carcasses than they were to explore healthy termites in the control set. Time spent antennating carcasses and mutilated termites were significantly longer compared to time spent antennating the control termites. The type of termite carcass encountered significantly influenced the frequency (*F* = 70.551; df = 6, 14; *P*<0.01) and time spent (*F* = 15.042; df = 6, 14; *P*<0.01) in grooming behavior by the workers. However, the HSD post-hoc test revealed that there was no significant difference in the frequency at which workers performed grooming behaviors on freshly killed and 1-day-old carcasses compared to control termites. In contrast, grooming of the mutilated termites by workers was significantly more common than grooming of any of the other sample types tested. Grooming constituted approximately 20.7±1.4% (*N* = 390) of total behaviors exhibited by workers exposed to mutilated termites. It is likely that the workers were checking the condition of the mutilated termites and then rejecting those that were heavily mutilated, as evidenced by the relatively high cannibalization rate (a total of 31 out of 60 [51.7%]) at the end of the experiment. The time spent grooming oven-killed carcasses was significantly higher than the time spent grooming the control termites.

**Table 1:** Behaviors (mean ± SE) of visible workers of *Coptotermes formosanus* in the first 15 min after contact with treatment individuals.

	Control	Freshly killed	1 day	3 days	7 days	Oven-killed	Mutilated
*Antennation*							
Frequency	0.217±0.014 a	0.894±0.158 b	0.876±0.046 b	0.181±0.026 a	0.333±0.022 a	1.167±0.209 b	0.408±0.053 a
Time spent	9.7±5.8 a	203.0±47.1 b	141.3±26.0 b	30.0±16.9 ac	86.7±8.1 bc	116.7±12.2 bc	78.7±15.7 bc
*Crawling dead body piles*							
Frequency	–	0.016±0.008 a	0.005±0.005 a	0.088±0.088 a	0.585±0.067 b	0.036±0.032 a	–
Time spent	–	2.7±1.5 a	1.0±1.0 a	25.3±25.3 a	158.7±34.3 b	3.3±2.8 a	–
*Active grooming*							
Frequency	0.271±0.036 ab	0.331±0.033 a	0.362±0.045 a	0.012±0.012 c	0.000±0.000 c	0.135±0.033 b	0.744±0.043 d
Time spent	49.3±28.5 ab	397.3±158.2 b	244.0±76.0 b	3.3±3.3 ac	0.0±0.0 c	341.0±48.0 b	104.0±29.5 b
*Dragging*							
Frequency	–	0.023±0.004 ab	0.016±0.002 a	0.006±0.006 a	0.000±0.000 a	0.048±0.012 b	0.009±0.003 a
Time spent	–	5.7±2.2 a	2.7±0.7 ab	0.7±0.7 bc	0.0±0.0 c	4.7±0.7 a	1.7±0.7 abc
*Dead body entombment*							
Frequency	–	0.000±0.000 a	0.000±0.000 a	0.018±0.011 a	0.022±0.006 a	0.012±0.012 a	–
Time spent	–	0.0±0.0 a	0.0±0.0 a	2.7±1.3 ab	2.1±0.1 b	1.0±1.0 ab	–
*Jerking*							
Frequency	0.000±0.000 a	0.000±0.000 a	0.024±0.009 a	0.031±0.017 a	0.024±0.010 a	0.202±0.047 b	0.088±0.028 a
Time spent	0.0±0.0 a	0.0±0.0 a	4.3±2.3 ab	7.0±6.0 ab	7.0±3.0 ab	19.7±2.7 b	17.0±6.1 b
*State of carcasses at 48 hours*							
	–	30–40% buried <40%cannibalized	30–40% buried	30–40% buried	30–40% buried	30–40% buried <40%cannibalized	<40%cannibalized

Means in the same row followed by same letters were not significantly different, *P*>0.05.

Frequency of carcass-burying behavior and time spent on this activity differed significantly among the different types of termite carcasses tested (frequency: *F* = 8.317; df = 5, 12; *P*<0.01; time: *F* = 10.631; df = 5, 12; *P*<0.05). The HSD post-hoc test revealed that freshly killed, 1-day-old, and oven-killed carcasses were more likely to be dragged either to the connecting tube or into the soil compared to the other carcass types tested. We interpreted this to be an act of entombment. More time was invested in stacking sand on 7-day-old carcasses than on the other types of termite carcasses tested (*F* = 5.246; df = 4, 10; *P*<0.05). In addition, the freshly killed and oven-killed carcasses were groomed and subsequently removed to the connecting tube to be eaten (Figure 1A).

The behavior of *C. formosanus* differed significantly among the types of termite carcasses tested in terms of the frequency and time spent crawling under the dead piles of bodies (frequency: *F* = 18.670; df = 4, 10; *P*<0.01; time: *F* = 4.440; df = 4, 10; *P*<0.01): The frequencies and time spent increased as the carcasses aged. Termites often crawled under the 7-day-old dead body piles, and this behavior was followed by carcass-burying activity without grooming of the carcasses.

The time invested in jerking was significantly longer when the termites encountered oven-killed carcasses and mutilated termites compared to when they encountered control (healthy) termites (*F* = 10.273; df = 6, 14; *P*<0.01).

### *R. speratus*

There was significant difference between the frequency of antennation among the killed, mutilated, and healthy termites (frequency: *F* = 9.686; df = 6, 14; *P*<0.01). However, the time spent in antennation was similar across carcasses tested (*F* = 2.297; df = 6, 14; *P*>0.05) (Table 2). In contrast, there were significant differences between the frequencies and time spent in grooming behaviors among the samples tested (frequency: *F* = 21.437; df = 6, 14; *P*<0.01, time: *F* = 13.694; df = 6, 14; *P*<0.05). The HSD post-hoc test revealed that the grooming frequency and time allocation was significantly greater for mutilated termites than for any of the other test samples; for mutilated termites it constituted 30.6±2.4% out of 428 behavioral acts within the 15 min observation period. However, grooming behavior time and frequency did not differ significantly among the control and the other sample types tested.

**Table 2:** Behaviors (mean ± SE) of visible workers of *Reticulitermes speratus* in the first 15 min after contact with treatment individuals.

	Control	Freshly killed	1 day	3 days	7 days	Oven killed	Mutilated
*Antennation*							
Frequency	0.685±0.028 a	0.827±0.086 ab	1.129±0.075 abc	1.449±0.192 bc	1.095±0.048 abc	0.758±0.081a	1.691±0.284 c
Time spent	36.0±4.7 a	32.0±18.7 a	76.7±23.8 a	77.0±22.9 a	26.7±8.0 a	28.3±9.8 a	81.3±1.3 a
*Crawling dead body piles*							
Frequency	–	0.200±0.200 a	0.269±0.005 ab	0.697±0.072 ab	1.101±0.107 b	0.000±0.000 a	–
Time spent	–	1.0±1.0 a	18.3±5.8 b	35.0±4.1 b	26.3±7.8 b	0.0±0.0 a	–
*Active grooming*							
Frequency	0.052±0.008 ab	0.290±0.054 ac	0.388±0.092 c	0.127±0.002 ab	0.000±0.000 b	0.000±0.000b	0.844±0.118 d
Time spent	10.7±1.3 ab	52.0±28.9 bc	112.0±47.7 bc	26.0±4.9 bc	0.0±0.0 a	70.7±40.4 bc	165.3±15.0 c
*Dragging*							
Frequency	–	0.036±0.022ab	0.137±0.025b	0.041±0.010ab	0.000±0.000a	0.000±0.000 a	0.380±0.057d
Time spent	–	2.7±2.2 ab	8.7±2.3 bc	2.0±0.0 ab	0.0±0.0 a	30.0±11.5 c	11.5±9.4 c
*Dead body entombment*							
Frequency†	–	0.007±0.007	0.007±0.007	0.000±0.000	0.000±0.000	0.000±0.000	–
Time spent†	–	0.7±0.7	0.3±0.3	0.0±0.0	0.0±0.0	0.0±0.0	–
*Jerking*							
Frequency†	0.000±0.000	0.000±0.000	0.036±0.028	0.000±0.000	0.000±0.000	0.000±0.000	0.000±0.000
Time spent†	0.0±0.0	0.0±0.0	2.0±1.2	0.0±0.0	0.0±0.0	0.0±0.0	0.0±0.0
*State of carcasses at 48hours*							
	–	30–40% buried <20%cannibalized	30–40% buried <20%cannibalized	70–80%buried	70–80%buried	30–40%buried <20%cannibalized	<20%cannibalized

Means in the same column followed by same letters were not significantly different, *P*>0.05.

† , Statistical analysis was not attempted because the dependent variables were constant in all cases.

The frequencies and time allocated to crawling under dead piles of bodies differed among the samples tested (frequency: *F* = 3.334; df = 4, 10; *P*<0.05; time: *F* = 28.399; df = 4, 10; *P*<0.01). There was a marked increase in the total number of acts (59.2±7.1%, *N* = 159) exhibited by workers towards 7-day-old carcasses compared with any other test sample. The durations of crawling under dead piles of bodies were significantly different for 1-, 3-, and 7-day-old carcasses when compared with freshly killed and oven-killed carcasses. Furthermore, the termites constructed shelter tubes that attached to or went through the body piles instead of only stacking sand on the surface of the piles.

**Figure 2:**
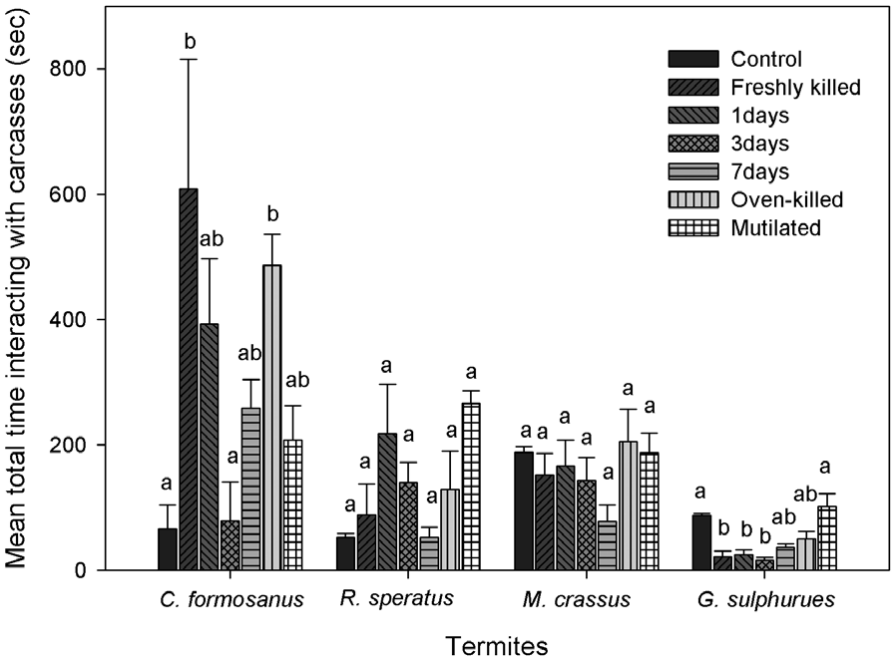
Mean total time (s) of termites interacting with carcasses. Error bars represent standard error of means. Means within a group followed by same letters were not significantly different, *P*>0.05.

The major differences between the behaviors of *R. speratus* and *C. formosanus* were that (1) dragging of mutilated termites into the connecting tube was exhibited only by *R. speratus*, particularly for mutilated termites (frequency: *F* = 62.952; df = 5, 12; *P*<0.01; time: *F* = 12.834; df = 5, 12; *P*<0.05) (Figure 1B), and (2) *R. speratus* fed on freshly killed and 1-day-old carcasses but *C. formosanus* only fed on freshly killed and oven-killed carcasses and mutilated termites.

### *M. crassus*

Overall, workers of this species performed relatively fewer behavioral acts compared to *C. formosanus* and *R. speratus* (Figure 1C). Significant differences in frequency of antennation (*F* = 3.186; df = 6, 32; *P*<0.05), grooming (*F* = 4.213; df = 6, 32; *P*<0.01), and dragging (*F* = 5.677; df = 5, 30; *P*<0.01) were shown by healthy workers toward carcasses and mutilated termites. Significant differences also were found in time allocation for grooming (*F* = 4.018; df = 6, 32; *P*<0.05), crawling under the dead piles (*F* = 3.475; df = 4, 25; *P*<0.05), and dragging (*F* = 8.203; df = 5, 30; *P*<0.05). In terms of frequencies and total time allocation, antennation was the most common behavior, followed by grooming in workers exposed to freshly killed and 1- and 3-day-old carcasses, oven-killed carcasses, and mutilated termites (Table 3).

**Table 3:** Behaviors (mean ± SE) of visible workers of *Microcerotermes crassus* in the first 15 min after contact with treatment individuals.

	Control	Freshly killed	1 day	3 days	7 days	Oven killed	Mutilated
*Antennation*							
Frequency	0.723±0.039 a	1.480±0.251 ab	1.486±0.263 ab	1.694±0.182 ab	0.997±0.166 a	1.259±0.159 ab	2.184±0.346 b
Time spent	111.7±31.6 a	87.5±20.5 a	78.5±14.3 a	91.8±17.2 a	58.5±27.5 a	94.7±23.1 a	91.8±19.0 a
*Crawling dead body piles*							
Frequency	–	0.000±0.000 a	0.078±0.011 a	0.095±0.007 a	0.037±0.004 a	0.000±0.000 a	–
Time spent	–	8.7±7.7 ab	5.0±1.7 ab	9.7±6.1 a	3.2±1.5 ab	0.0±0.0 b	–
*Active grooming*							
Frequency	0.095±0.008 a	0.306±0.071 ab	0.285±0.092 ab	0.113±0.019 a	0.000±0.000 a	0.406±0.124 ab	0.550±0.079 b
Time spent	58.7±5.8 a	50.0±12.3 ab	79.3±29.9 ab	35.3±18.7 ab	0.0±0.0 b	102.0±36.9 a	90.7±15.4 a
*Dragging*							
Frequency	–	0.035±0.022 ab	0.000±0.000 a	0.000±0.000 a	0.000±0.000 a	0.064±0.003 b	0.000±0.000 a
Time spent	–	2.0±1.2 ab	0.0±0.0 a	0.0±0.0 a	0.0±0.0 a	3.2±0.8 b	0.0±0.0 a
*Dead body entombment*							
Frequency†	–	0.000±0.000	0.000±0.000	0.000±0.000	0.000±0.000	0.000±0.000	–
Time spent†	–	0.0±0.0	0.0±0.0	0.0±0.0	0.0±0.0	0.0±0.0	–
*Jerking*							
Frequency	0.049±0.009a	0.144±0.077 a	0.060±0.014 a	0.114±0.021 a	0.039±0.001 a	0.095±0.017 a	0.187±0.066 a
Time spent	7.3±1.3 a	4.2±1.8 a	3.2±0.9 a	6.3±1.9 a	2.2±1.0 a	5.5±1.1 a	4.5±1.9 a
*State of carcasses at 48 hours*							
	–	>90%ignored	>90%ignored	>90%ignored	>90%ignored	>90%ignored	40–50%buried <20%cannibalized

Means in the same column followed by same letters were not significantly different, *P*>0.05.

† , Statistical analysis was not attempted because the dependent variables were constant in all cases.

The act of carcass dragging was occasionally performed on freshly killed and oven-killed carcasses. Although the behavior of crawling under the piles of dead bodies was not frequently encountered, termites frequently walked on the 1-, 3-, and 7-day-old carcass piles. This behavior, however, was not included in our analysis because it did not fit into the definition of carcass-burying behavior. In all instances, the carcasses were ignored and left unburied. In addition, sand barriers were constructed to block the access tubes to the containers. Mutilated termites were fed upon, and some were entombed in the connecting tube.

### *G. sulphureus*

Antennation was commonly observed when *G. sulphureus* workers came into contact with carcasses and mutilated termites. The frequency and time allocated to antennation by workers differed significantly between carcasses and mutilated termites (frequency: *F* = 12.608; df = 6, 32; *P*<0.01; time: *F* = 4.215; df = 6, 32; *P*<0.01). The HSD post-hoc test showed that antennation was more frequently performed on the mutilated termites and 7-day-old carcasses compared to the other types of carcasses, but mutilated termites were antennated for a longer period of time (Table 4). After antennating the piles of carcasses (Figure 1D), workers left the arena and did not return within the 15 min observation period.

**Table 4:** Behaviors (mean ± SE) of visible workers of *Globitermes sulphureus* in the first 15 min after contact with treatment individuals.

	Control	Freshly killed	1 day	3 days	7 days	Oven killed	Mutilated
*Antennation*							
Frequency	0.217±0.011 a	0.312±0.070 a	0.486±0.122 ab	0.345±0.156 a	1.820±0.498 bc	0.621±0.059 ab	2.074±0.520 c
Time spent	72.7±6.4 ab	21.5±9.0 a	23.5±7.0 a	16.3±4.0 a	36.2±5.9 ab	48.8±11.2 ab	101.8±20.8 b
*Crawling dead body piles*							
Frequency†	–	0.000±0.000	0.000±0.000	0.000±0.000	0.000±0.000	0.000±0.000	–
Time spent†	–	0.0±0.0	0.0±0.0	0.0±0.0	0.0±0.0	0.0±0.0	–
*Active grooming*							
Frequency†	0.012±0.009	0.000±0.000	0.000±0.000	0.000±0.000	0.000±0.000	0.000±0.000	0.000±0.000
Time spent†	13.3±9.6	0.0±0.0	0.0±0.0	0.0±0.0	0.0±0.0	0.0±0.0	0.0±0.0
*Dragging*							
Frequency†	–	0.000±0.000	0.000±0.000	0.000±0.000	0.000±0.000	0.000±0.000	0.000±0.000
Time spent†	–	0.0±0.0	0.0±0.0	0.0±0.0	0.0±0.0	0.0±0.0	0.0±0.0
*Dead body entombment*							
Frequency†	–	0.000±0.000	0.000±0.000	0.000±0.000	0.000±0.000	0.000±0.000	–
Time spent†	–	0.0±0.0	0.0±0.0	0.0±0.0	0.0±0.0	0.0±0.0	–
*Jerking*							
Frequency	0.002±0.002 a	0.000±0.000 a	0.034±0.003 b	0.000±0.000 a	0.022±0.001 ab	0.012±0.012 ab	0.000±0.000 a
Time spent	0.7±0.7 a	0.0±0.0 a	1.2±0.5 a	0.0±0.0 a	0.0±0.0 a	0.8±0.8 a	0.0±0.0 a
*State of carcasses at 48 hours*							
	–	>90%buried	>90%buried	>90%buried	>90%ignored	>90%buried	*>90%buried or >90%ignored

Means in the same column followed by same letters were not significantly different, *P*>0.05.

† , Statistical analysis was not attempted because the dependent variables were constant in all cases.

* , The carcasses were either buried or ignored in some replicates.

## Discussion

The exact mechanisms by which the presence of carcasses and mutilated nestmates recruit termites are not fully understood, but the process seems to be mediated by chemical stimuli from the carcasses, which can be detected by the insects' chemosensory sensilla (e.g., antennae, maxillary and labial palps) and tactile stimuli [21]. A total of 51 compounds from the integument of a dead alate termite were extracted, and the majority contained fatty acid, phenol, and indol [13], [16]. The combination of the compounds was shown to trigger carcass-burying behavior in dealates of *P. spiniger*. Fatty acids (oleic and linoleic acids) that act as a necromone (death-recognition cue) have been identified in ants [1], [2], [3], [19], cockroaches [22], bees [23], [24], and social caterpillars [25]. Beside chemical stimuli, tactile stimuli is also required to elicit carcass burial activity in *Reticulitermes virginicus* (Banks) [21].

In general, intact conspecific carcasses were thought to elicit weaker responses from healthy nestmates compared to crushed carcasses [25]. However, this notion is not supported by the data from the oven-killed carcasses (with an intact body) from the present study. The frequency and time spent on certain behavioral acts was significantly greater when resident termites encountered oven-killed carcasses than when they encountered crushed or control termites. Fatty acids are known to be enzymatically derived from dead or injured cells via the breakdown of triglycerides in the insect body. By exposing termites to temperatures of 55–65°C for 20 min, the enzymes involved in the oxidation process would be denatured. Hence, the level of fatty acid on the body surface of oven-killed termites would theoretically remain unchanged or low. This premise is further supported by Akino and Yamaoka [26], who reported that the fatty acid level on the body surface of *Formica japonica* Motschoulsky did not increase after it was irradiated. Recently, death-recognition behavior was reported to be triggered by the fading of chemical signals from the cuticle of the ant *Linepithema humile* (Mayr) instead of by fatty acids [27]. To date, this phenomenon has been reported only in ants. However, in termites this possibility cannot be completely ruled out. Chemical analysis is warranted to identify the chemical(s) that are involved as death-recognition cues for termites. An alternative explanation for the inconsistent result with those crushed carcasses is that the encounter of heat-killed carcass may be novelty to termites. Termites have adaptations to avoid heat injury, for example, the modification in nest architecture [28] and foraging patterns [29] in response to climatic changes. This explains why heat-killed carcasses are not readily encountered by termites compared to the dead mutilated conspecific. Thus more time and efforts were spent by the termites to investigate the threats posed by these heat-killed carcasses.

In insects, alarm pheromones are regularly released to induce necessary behavioral responses in the presence of danger (e.g., defensive behavior, aggressive behavior, evacuation/dispersal response, aggregation behavior, and recruitment). In termites, detailed studies of the alarm pheromones and secretions have been conducted [30], [31]. The alarm responses were characterized as repeated forward-backward jerking and rapid walking to alert and attract other members of the group [32], [33]. At present, however, nothing is known about alarm pheromones in worker termites. The present study showed that mutilated live termites were constantly groomed by their nestmates. Ultimately, these termites were fed upon because the hemolymph from the wound induced cannibalism [34]. We speculate that the mutilated workers may have released alarm pheromones that were attractive to healthy nestmates.

To our knowledge, this is the first documentation of termites crawling under aging carcass piles, although Saran and Rust [11] have previously reported that healthy *Reticulitermes hesperus* Banks showed no tendency to avoid 7-day-old termite carcasses. Moreover, the level of oleic acid has been shown to increase on the body surface of test carcasses (e.g., *F. japonica* [26], *Oniscus asellus* Linn [25]) as they age. Thus, the existence of this behavior in our study might be explained by an increased level of necromones in the aged carcasses that might be highly attractive to termites.

*C. formosanus* and *R. speratus* often walled off the old carcasses (3- and 7-day-old carcasses) with sand at the spot where the carcasses were introduced. Similar observation was also made in *R. virginicus* where workers were found to carry more sand to wood blocks that containing carcasses for carcass burial activity [21]. In contrast, newly killed termites and mutilated live termites were intensively carried into the soil or connecting tubes to be fed upon. This suggests that only newly killed termites are consumed as a food source [15], [35], whereas carcasses older than 3 days are not acceptable as food. This raises interesting questions about whether termites exhibit the same behavior as the ant *T. lichtensteini*: This ant species handled old carcasses by transporting them outside the nests, whereas new carcasses were buried [20]. If this behavior exists in termites, the chemical signatures that are involved in stimulating such behaviors remain to be determined.

In the present study, we documented the undertaking behaviors (i.e., carcass-burying behavior, necrophagy, and cannibalism) that were performed by a given termite species on certain types of nestmate carcasses or mutilated termites. Such behavioral responses likely are associated with ecological adaptations that are of prime importance to colony fitness. Factors influencing the behaviors are threefold. The first is the threat posed to the resident colony by different types of carcasses. We speculate that a series of behavioral acts performed towards termite carcasses might be a way of assessing the threat level (i.e., how many carcasses are there? Are any individuals in the pile still alive? Do they pose a threat to the colony?). The results of this study suggest that *C. formosanus* spent more effort investigating (antennating and grooming) freshly killed and oven-killed carcasses (Figure 2), whereas those that were dead longer (3- and 7-day-old carcasses that were overgrown with a fast-growing fungus) were given a cursory examination and then buried at once. The burial behavior is central to preventing the transmission of pathogenic microbes among colony members [16]. In the present study, mutilated termites were highly groomed and dragged into tunneling tubes by nestmates (particularly for *R. speratus*). This behavior also was observed in a study of the fungus-growing termite *P. spiniger* [16] and in *Macrotermes carbonarius* (Hagen), and *Macrotermes gilvus* (Hagen) during field trips (Neoh and Lenz, unpubl. data). On several occasions during the field trips, we carefully picked up termite foragers using clean forceps (without causing injuries), kept them in Petri dishes for about 1 min, and then released them back to the foraging trails. The disturbed termites were immediately attended and groomed by other foragers and eventually were carried to the nest. In this context, the mutilated termites likely were examined for injuries and smeared with saliva that contains antiseptic properties [36]. In contrast, limited behavioral acts and time were spent by *M. crassus* and *G. sulphureus* regardless the natures of the carcasses (Figure 2). These termites seemingly searched for survivors and then blocked off (*M. crassus*) or abandoned (*G. sulphureus*) the area during the initial 15 min observation.

The second factor influencing the behaviors relates to feeding habits and dietary input of nitrogen. *C. formosanus* and *R. speratus* are wood feeders that readily attack living trees and wooden structures that contain little nitrogen [37]. Thus, the limited nitrogen in the termites' diet likely triggers necrophagy or cannibalism. The uric acid in termite carcasses can be degraded by the gut bacteria of nestmates, thereby providing a nitrogen source [38]. In a termite starvation study using laboratory groups of Formosan termites, cannibalism and necrophagy were intensive under starvation and nutrient deficiency conditions [15]. However, this phenomenon is limited to relatively fresh carcasses; aged carcasses that are covered with microbial and fungal growth are not consumed [16]. Unlike the former species, *G. sulphureus* and *M. crassus* are plant litter foragers that forage on leaf litter and wood litter [37]. Numerous studies have shown that the nitrogen content of decomposed wood is higher than that of in non-decayed wood [39], [40], [41]. Hence, for these species, obtaining nitrogen from termite carcasses might be unnecessary, and in fact these species exhibited a low rate of necrophagy in the present study. These feeding habits likely explain why carcass-burying activity was commonly observed but cannibalism or necrophagy was rarely seen for *M. crassus* and *G. sulphureus*.

**Figure 3:**
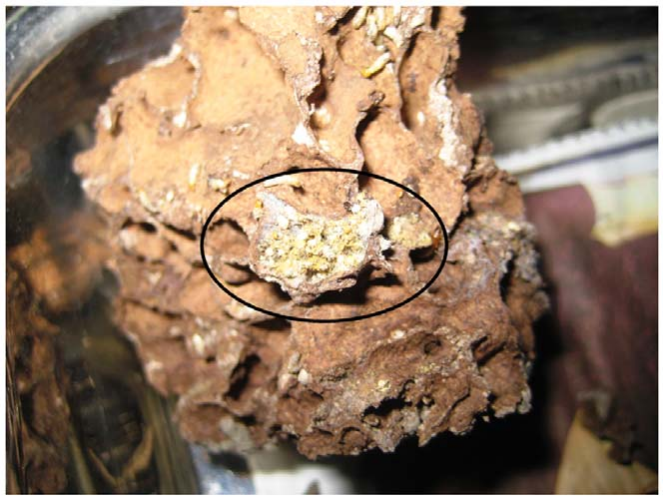
The outcome of undertaking performed by *Microcerotermes crassus* in a dissected nest. The carcasses (circled) apparently were confined in an isolated cavity with the wall sealed off from the rest of the nest.

Finally, the behavioral responses of termites encountering carcasses likely evolved in response to nesting ecology. *R. speratus and C. formosanus*, which nest within food sources (e.g., old and damaged trees), either consumed or actively entombed their dead, as would be necessary within the more open structure of their living/feeding area. In contrast, *M. crassus* and *G. sulphureus* construct discreet carton nests and forage outside the nest [42]. Thus, these species exhibited minimal handling of carcasses and blocking off the tunnel containing the carcasses within the nests (*M. crassus*) (Figure 3) or avoidance of foraging areas containing dead termites (*G. sulphureus*) [43].

### Summary and Conclusion

In general, *G. sulphureus* demonstrated avoidance behavior (or necrophobia) by having limited contact with carcasses, and *M. crassus* did so by blocking off areas in the access tubes that contained carcasses. In contrast, *C. formosanus* and *R. speratus* performed relatively more behavioral acts towards carcasses. The behaviors of the latter two species cast a new light on the earlier study conducted by Su et al. [5], who reported that termites are necrophobic in nature. Other studies also have disputed this claim. For example, Campora and Grace [10] suggested that the avoidance of borate-treated wood by *C. formosanus* occurred not because of necrophobia but because the high mortality of the foraging workers reduced the pheromone trail. The premise of necrophobia was further disproved in a study of the termiticide fipronil. Hu et al. [12] found that fipronil-treated termite carcasses were neither expelled nor walled off with sand but instead were continuously groomed by healthy nestmates.

Though heat-killed termites may be novel (i.e. differ in some way from those they would ordinarily encounter in nature), the types of carcasses used in this study likely simulate situations that the termites would face in nature, such as aggressive interactions between colonies, a diseased colony, accidental damage to nest or foraging trails, and predator invasions into a colony, all of which might lead to the presence of nestmate carcasses. However, we would expect to observe different behavioral acts by resident termites when encountering a pile of dead heterospecific or non-nestmate conspecific termites. In their study of *M. crassus*, Wong and Lee [44] found that non-nestmates carcasses were buried instead of the accessing tubes being blocked off, as the latter behavior was observed among nestmates in the present study. Similarly, *T. lichtensteini* workers acted differently when encountering heterospecific workers [20]. In light of the results of the present study, we propose that the avoidance response exhibited by subterranean termites when exposed to carcasses depends on the nature of the carcasses and the termite species involved. The reaction of termites towards nestmate carcasses is thus more complex than was previously thought.

## Materials and Methods

### Termite species

*C. formosanus* has been cultured in the laboratory of the Research Institute for Sustainable Humanosphere of Kyoto University since the early 1990s. It originated from Wakayama Prefecture. The colony was maintained at 28±2°C and >80% relative humidity (RH). Samples of *R. speratus* were collected from a heavily infested tree branch at the Uji Campus of Kyoto University, Japan. Samples of two Southeast Asian mound-building termite species, *M. crassus* and *G. sulphureus*, were collected from the road side along Sultan Azlan Shah Street in Penang, Malaysia. For the latter two species, two field colonies for each species were tested. The mature nests of *G. sulphureus* chosen ranged from 0.3 to 0.5 m in height. They were carefully excavated to remove the outer layer, and only a partial of ball of vegetation (i.e., food lumps), which housed the termites, was sampled. For *M. crassus*, the entire arboreal nest was removed. No special permits were required for the described field collection. The collection sites also were not privately owned and the collection did not involve endangered or protected species. The nests were transported to the laboratory, and the termites were harvested by tapping the detached nest materials/food lumps onto trays. Parts of the *M. crassus* nests that contained dead or injured termites caused by termite sampling were kept under laboratory conditions for 7 days. The nests were then cut open to investigate the distribution of the carcasses in the nest structure.

### Experimental set up

**Figure 4:**
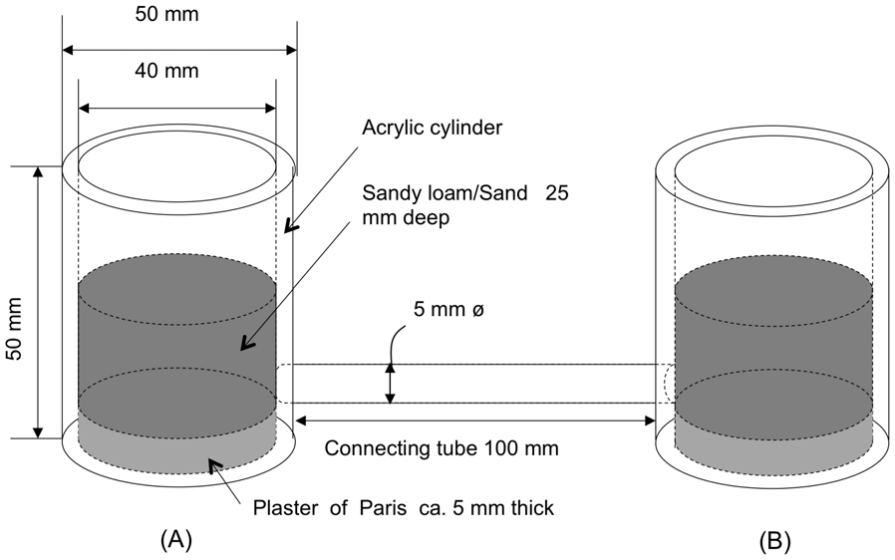
Experimental set up for examining the effects of dead bodies and mutilated termites on healthy worker termites.

A test unit consisted of two containers (inner diameter: 40 mm; height: 50 mm) connected by an acrylic tube (inner diameter: 5 mm; length: 100 mm) (Figure 4). The bottom of the containers was layered with plaster of Paris (GC Corporation, Tokyo, Japan), which was covered by a 25 mm thick layer of 20% water content sandy loam (for *C. formosanus* and *R. speratus*) or sand sieved through 40 mesh with 20% water content (for *G. sulphureus* and *M. crassus*) to provide humidity.

In another *Reticulitermes* species, carcass-burying activity was mostly performed by old workers [14]. Thus, in these experiments, for each species tested we used 100 old workers, as determined by the presence of a gut that contained wood and a mandible and body that were highly sclerotized [45], [46]. We assumed that the termites would perform the undertaking task equally. Soldiers were added based on the worker∶ soldier ratio from the field populations: 100 workers∶ 2 soldiers for *R. speratus* [47] and *M. crassus* [48] and 100 workers∶ 10 soldiers for *C. formosanus* [49] and *G. sulphureus* [50]. Occasionally, soldiers appeared in the observing arena, but the undertaking activity was taking place regardless the presence of soldiers. Thus, we singled out the role of soldier in the present study. The test termites (designated as resident workers) were introduced into test container A (Figure 4) and acclimatized for 1 day prior to testing in order to allow termites to tunnel to container (B) and become evenly distributed in the set up.

### Dead or mutilated test samples

Test samples of dead or mutilated nestmates were prepared as follows: (a) Crushed termites: Termites were crushed to death using soft forceps that make the termite's body fluid oozed out and covered the body parts. Crushed carcasses were kept in covered Petri dishes under laboratory conditions during the aging process. Freshly killed, 1-, 3-, and 7-day-old carcasses were tested. Generally, 3- and 7-day-old carcasses were overgrown with a fast-growing fungus during the experiment; (b) Oven-killed termites: Termites were killed in an oven at temperatures ranging from 55 to 65°C for 20 min; these carcasses had an intact body, but sometimes a little body fluid appeared at the tip of abdomen; (c) Mutilated termites: Either the left or right middle and hind legs of the termites were removed; and (d) Healthy termites were used as a control. We marked the termites in groups (c) and (d) with a dot of black ink (Sharpie, Oak Brook, IL, USA) on the head capsule to differentiate them from the resident workers. Laboratory tests prior to the experiments showed that the ink caused no significant behavioral changes or mortality in termites. Approximately 20±2 termites of each type (a, b, c, and d) were used in each test.

### Behavioral assay

Behavioral assays were conducted after the 1 day period of acclimatization to the laboratory conditions (28±2°C, >80% RH, and in complete scotophase). The termites were acclimatized under light phase for 30 min before the test samples were introduced into container B (Figure 4). Because the time that termites took to approach the test samples varied (see Results), we began video recording (Sony® DRC-TRV340E, Tokyo, Japan) when worker termites first contacted the test samples. Video recording was conducted for 15 min. All behaviors exhibited during the 15 min video recordings were identified and categorized as exploratory behavior, grooming, carcass-burying behavior, and/or alarming behavior, as described by Crosland et al. [51] with slight modifications (Table 5). Next, direct observations (∼1 min with the naked eye) were made at selected time intervals (i.e., 30 min, 1, 2, 3, 4, 8, and 24 h) to see if any carcass-burying activity, etc. had occurred. Final observations were recorded at 48 h. The experimental set ups were dismantled and direct counting of carcasses was conducted. Overall, three methods of carcass handling were commonly observed after 48 h: They were buried, cannibalized, or ignored. The number of carcasses handled was then counted and rated in percentage. The tests were replicated three times for each type of dead or mutilated nestmate. For each replicate, new groups of 100 old workers (resident workers) and 20±2 new freshly killed, 1-, 3-, and 7-day-old, oven-killed, and mutilated samples were used.

**Table 5:** Behavioral repertoire shown in the first 15 min of interactions of visible workers of *Coptotermes formosanus*, *Reticulitermes speratus*, *Microcerotermes crassus*, and *Globitermes sulphureus* with 1-, 3-, 7-day-old and oven-killed carcasses and mutilated termites.

Behaviors	Definitions
*Exploratory behavior*	
Antennation	Worker termites use their antennae to touch dead or mutilated termites for up to 3 sec
Crawling under dead body piles	Termites burrow down beneath and crawl under piles of dead bodies repeatedly
*Grooming*	
Active grooming	Termites lick the body of dead termites (i.e., mouth, abdominal, or anal parts)
*Carcass-burying behavior*	
Dragging	Termites use mouth parts to carry the termite bodies
Dead body entombment	Termites carry sand particles and place them on termite bodies
*Alarming behavior*	
Jerking	A repeated rapid body movement that moves the body to the front and back

### Data analysis

Only the termites that appeared in the observing arena where termite carcasses were placed (visible termites) were taken into account for analysis. Mean frequency of individuals showing a specific behavioral response was unable to be generated as tracking on an individual is not possible in this experimental design. Thus, mean frequency of a particular behavior exhibited by a visible worker, that represents how much effort is spent by visible termites in handling termite dead body within the 15 min observation period was calculated as F = B/T, where B = the number of occurrences of a particular behavior and T = the number of visible termites that appeared on the surface of container B during the first 15 min (adopted from Crosland et al. [14] with slight modification). The frequencies were then subjected to Log (n+0.5) transformation to produce normality of the distribution. The data were separated by carcass type (a, b, c, and d) and compared among these types for each species of termite tested using multivariate analysis of variance (MANOVA) and separated with Tukey's HSD (SPSS, v.11.0, SPSS Inc., Chicago, IL).
